# Supplementary material for: Synthesis, Characterization and Anticancer Efficacy Studies of Iridium (III) Polypyridyl Complexes against Colon Cancer HCT116 Cells
Source: Molecules. 2022 Aug 25;27(17):5434. doi: 10.3390/molecules27175434 (PMC9458069; doi:10.3390/molecules27175434)
Supplement: Supplementary file 1 [file molecules-27-05434-s001.zip › molecules-1850515-supplementary.pdf]

molecules

MDPI

Supplementary Materials

Synthesis, Characterization and Anticancer Efficacy Studies of Iridium (III) Polypyridyl Complexes against Colon Cancer HCT116 Cells

Biao Xie [1,2], Yi Wang [3], Di Wang [2], Xingkui Xue [4] and Yuqiang Nie [1,5,*]

[1] The First Affiliated Hospital, Jinan University, Guangzhou 510630, China
[2] Department of Gastroenterology, People's Hospital of Longhua, Shenzhen 518109, China
[3] School of Pharmacy, Guangdong Pharmaceutical University, Guangzhou 510006, China
[4] Department of Medical Research Center, People's Hospital of Longhua, Shenzhen 518109, China
[5] Department of Gastroenterology, School of Medicine, Guangzhou First People's Hospital, South China University of Technology, Guangzhou 511458, China
* Correspondence: nieyq@medmail.com.cn

Citation: Xie, B.; Wang, Y.; Wang, D.; Xue, X.; Nie, Y. Synthesis, Characterization and Anticancer Efficacy Studies of Iridium (III) Polypyridyl Complexes against Colon Cancer HCT116 Cells. *Molecules* **2022**, *27*, 5434. https://doi.org/10.3390/molecules27175434

Academic Editor: Mei-Chin Lu

Received: 20 July 2022
Accepted: 20 August 2022
Published: 25 August 2022

Publisher's Note: MDPI stays neutral with regard to jurisdictional claims in published maps and institutional affiliations.

Copyright: © 2022 by the authors. Licensee MDPI, Basel, Switzerland. This article is an open access article distributed under the terms and conditions of the Creative Commons Attribution (CC BY) license (https://creativecommons.org/licenses/by/4.0/).

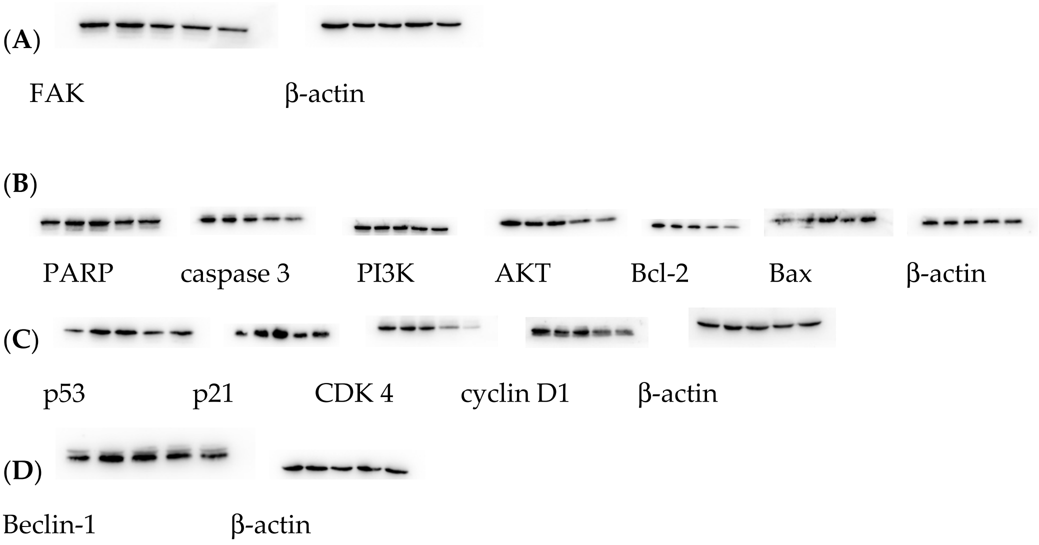

Figure S1. Gel pictures of the performed experiments. (**A**) Wound healing experiment. (**B**) Apoptotic experiment. (**C**) Cell cycle arrest. (**D**) Autophagy.

.
